# Seven years since defining the top five research priorities in physician-provided pre-hospital critical care – what did it lead to and where are we now?

**DOI:** 10.1186/s13049-018-0562-6

**Published:** 2018-11-20

**Authors:** Kristi G. Bache, Marius Rehn, Julian Thompson

**Affiliations:** 1 The Norwegian Air Ambulance Foundation, Oslo, Norway; 2 Institute of Basic Medical Science, University of Oslo, Oslo, Norway; 3 Faculty of Health Sciences, University of Stavanger, Stavanger, Norway; 4 Division of Emergencies and Critical Care, Department of Anaesthesiology, Oslo University Hospital, Oslo, Norway; 5 Adult Intensive Care Unit, North Bristol NHS Trust, Bristol, UK; 6 Great Western Air Ambulance, Bristol, UK; 7 Severn Major Trauma Network, Bristol, UK

In trying to match limited resources to the research fields most critically in need, it seems reasonable to start the process by asking the right questions. In October 2011, Fevang et al. published a consensus process in the journal that defined the five most urgent topics to be prioritized for research within the field of physician-provided pre-hospital critical care [1]. The requirement was brought about by the lack of evidence-base for current pre-hospital practice and even for the utilization of physicians in this phase of care. To address these central issues, research leaders from physician-provided pre-hospital critical care systems across Europe were brought together in a consensus process with the aim of defining the most important research priorities in this rapidly evolving field of medicine. This Editorial introduces a series of articles that review what progress has been made in each of these 5 prioritized areas of pre-hospital research and reveals where the gaps in our knowledge still exist.

The controversy surrounding physician-manned emergency services operating in the pre-hospital phase is intriguing, as there seems to be a common understanding regarding the need for physicians in the emergency management of critically ill patients in the hospital [2–4]. Conversely, it could be seen as negligent to deprive patients of the physician skill set and diagnostic capability if available. The concept of delivering pre-hospital physicians to the moment of greatest patient need evolved in Scandinavia more than 60 years ago. However, the vast majority of emergency patients are safely cared for by ambulance personnel or nurses before they arrive at the hospital. This may be partly explained by the extraordinary range of acuity and conditions that emergency services are required to respond to. Whilst a non-physician response may be appropriate in many cases, limited resources and the increased costs of advanced care undoubtedly plays an important role in the decision-making and availability of staff. Therefore, evidence-based decision-making for the dispatch of emergency medical services and delivery of advanced interventions is of central importance, balancing the level of safe care appropriate to a patient’s need with resource availability and cost benefit. This is a fine balance, demanding reasoning based on evidence and experience, systematically collected and analyzed, in order to increase the chances of making the right decision.

The 2011 expert panel proposed that these subjects of staffing, dispatch, advanced interventions such as ultrasound and airway management and timely delivery of care were the research priorities in physician-provided pre-hospital emergency service. As a call to action, these topics were promoted as the most important priorities in elucidating how resources are best administered, what the role of the pre-hospital physician is, and what are the implications of advanced interventions made possible by the presence of the physician.

In reviewing progress we find it pertinent to ask, not simply if the questions have been answered – but have they even been addressed? Are we closer to finding the answers, and have the quality of the literature on the topic of physician provided pre-hospital care improved and the results become more consistent? Defining research priorities does not mean we can sit back and wait for the results. The monitoring and evaluation of progress is crucial, as good intentions need caring for, and firm guidance by focus of effort on specific questions.

The top five research priorities in physician-provided pre-hospital critical care did not deliver an easy way out. The most controversial topics were, not surprisingly, brought forward by the expert panel as the most imperative to find answers to. In *appropriate staffing in pre-hospital critical care,* evidence for the role of the physician was examined. This question remains elusive despite numerous publications on the topic both before and after the priorities were published [5]. Some conditions, like traumatic brain injury, depend on early support of vital functions for favorable outcome [6], and the positive role of the physician may be reflected in decreased incidence of hypoxia, more normotension and improved neurological outcome [7]. But direct evidence linking patient outcome to the presence of the specific staff is multifactorial and requires more evidenced. *Pre-hospital advanced airway management* has been investigated on a large number of patients, but support in the literature for a positive effect on mortality has not been demonstrated [8]. It is a challenging topic to investigate, as modifying such a critical intervention in the name of research may be questionable. Agreement is needed on what is measured including outcome measures as using mortality alone may not lead to advance our knowledge? *Time windows for key critical interventions* and the influence of definitive in-hospital care on pre-hospital decisions is the third topic on the list. It is in the interest of the patient to receive advanced and complex interventions in a challenging environment if it improves outcome. Not if the risk of failing is too high, or the outcome is significantly improved by rapid transport to the hospital. Where do we draw the line and with what measures – Risk? Benefit? Time?? In examining the role of *pre-hospital ultrasound* the possibilities of using point of care diagnostics are addressed. Questions like which examinations can be readily transferred to the pre-hospital setting and how it can affect the patient pathway are highly relevant. With the emerging of new technology and more compact devices the introduction of these developments to pre-hospital setting needs standardization of the risks and benefits. Finally, *dispatch criteria for pre-hospital critical care* was put on the list, as it all begins with the activation of resources. In doing so, high-resource services are considered, and the acuity of the patients’ needs evaluated often under time critical conditions and with information from various sources.

Considering the challenges and impediments associated with the path towards an evidence-based practice in pre-hospital critical care, the great effort put in from numerous clinicians and scientists should be recognized. Progress is being made in investigating feasibilities of interventions, developing standard operating procedures, measuring outcomes, developing systems of care and patient safety in the pre-hospital care of patients. In this increasing flow of information, it is our responsibility to assess and evaluate what is known, to continue building on what is scientifically sound, clinically relevant, and make use of the evidence that already exists.

We have invited researchers and clinicians within the respective fields of the top five research priorities in physician-provided pre-hospital critical care, to summarize the knowledge that has been added since the priorities were defined, in up-to-date systematic review articles. With this we hope to bring forward an overview of what has been added and where we stand. We hope that this can contribute to moving physician provided pre-hospital care forward. What has and what hasn’t been addressed? for what reasons? and what is the relevance, actuality and utility of the priorities today? Are they still the top five? Did these questions define where the most benefit could be delivered to our patients? Has the time come for re-thinking and revising the priorities or are we on track towards future research for improved practice and a strengthened *out-of-hospital chain of survival* for the acutely ill or traumatized patient?

## References

[1] Fevang E, Lockey D, Thompson J, Lossius HM, Torpo Research C (2011). The top five research priorities in physician-provided pre-hospital critical care: a consensus report from a European research collaboration. Scand J Trauma Resusc Emerg Med..

[2] O'Malley AS, Draper DA, Felland LE (2007). Hospital emergency on-call coverage: is there a doctor in the house?. Issue Brief Cent Stud Health Syst Change.

[3] McConnell KJ, Johnson LA, Arab N, Richards CF, Newgard CD, Edlund T (2007). The on-call crisis: a statewide assessment of the costs of providing on-call specialist coverage. Ann Emerg Med.

[4] Jensen SM, Do HQ, Rasmussen SW, Rasmussen LS, Schmidt TA (2015). Emergency team calls for critically ill non-trauma patients in the emergency department: an observational study. Scand J Trauma Resusc Emerg Med..

[5] Sollid SJM, Rehn M (2017). The role of the anaesthesiologist in air ambulance medicine. Curr Opin Anaesthesiol.

[6] Boer C, Franschman G, Loer SA (2012). Prehospital management of severe traumatic brain injury: concepts and ongoing controversies. Curr Opin Anaesthesiol.

[7] Pakkanen T, Kamarainen A, Huhtala H, Silfvast T, Nurmi J, Virkkunen I (2017). Physician-staffed helicopter emergency medical service has a beneficial impact on the incidence of prehospital hypoxia and secured airways on patients with severe traumatic brain injury. Scand J Trauma Resusc Emerg Med.

[8] Fevang E, Perkins Z, Lockey D, Jeppesen E, Lossius HM (2017). a systematic review and meta-analysis comparing mortality in pre-hospital tracheal intubation to emergency department intubation in trauma patients. Crit Care.

